# Quality by Design-Driven Zeta Potential Optimisation Study of Liposomes with Charge Imparting Membrane Additives

**DOI:** 10.3390/pharmaceutics14091798

**Published:** 2022-08-26

**Authors:** Zsófia Németh, Ildikó Csóka, Reza Semnani Jazani, Bence Sipos, Henrik Haspel, Gábor Kozma, Zoltán Kónya, Dorina Gabriella Dobó

**Affiliations:** 1 Faculty of Pharmacy, Institute of Pharmaceutical Technology and Regulatory Affairs, University of Szeged, 6, Eötvös Street, H-6720 Szeged, Hungary; 2 Department of Applied and Environmental Chemistry, Faculty of Science and Informatics, Institute of Chemistry, University of Szeged, 1, Rerrich Béla Sqare, H-6720 Szeged, Hungary

**Keywords:** liposome, zeta potential, factorial design, optimisation, stearylamine, dicetyl phosphate

## Abstract

Liposomal formulations, as versatile nanocarrier systems suitable for targeted delivery, have a highly focused role in the therapy development of unmet clinical needs and diagnostic imaging techniques. Formulating nanomedicine with suitable zeta potential is an essential but challenging task. Formulations with a minimum ±30 mV zeta potential are considered stable. The charge of the phospholipid bilayer can be adjusted with membrane additives. The present Quality by Design-derived study aimed to optimise liposomal formulations prepared via the thin-film hydration technique by applying stearylamine (SA) or dicetyl phosphate (DCP) as charge imparting agents. This 3^2^ fractional factorial design-based study determined phosphatidylcholine, cholesterol, and SA/DCP molar ratios for liposomes with characteristics meeting the formulation requirements. The polynomials describing the effects on the zeta potential were calculated. The optimal molar ratios of the lipids were given as 12.0:5.0:5.0 for the *SA-PBS pH 5.6* (optimised sample containing stearylamine) and 8.5:4.5:6.5 for the *DCP-PBS pH 5.6* (optimised sample containing dicetyl phosphate) particles hydrated with phosphate-buffered saline pH 5.6. The *SA-PBS pH 5.6* liposomes had a vesicle size of 108 ± 15 nm, 0.20 ± 0.04 polydispersity index, and +30.1 ± 1.2 mV zeta potential, while these values were given as 88 ± 14 nm, 0.21 ± 0.02, and −36.7 ± 3.3 mV for the *DCP-PBS pH 5.6* vesicles. The prepared liposomes acquired the requirements of the zeta potential for stable formulations.

## 1. Introduction

### 1.1. Liposomal Formulations

Liposomal formulations, lipid bilayer-built up nanocarriers, provide a modern and innovative way for drug delivery. Liposomal administration of the active pharmaceutical ingredients (API) can reduce potential side effects and provide more favourable pharmacokinetic profiles and targeted therapy [1]. The typically negatively charged or neutral, nonpolar carbon chains of the wall forming phospholipids are oriented towards each other, while the polar heads condense into a layer along with the outer and inner aqueous phases. Due to this structural design, the vesicles can encapsulate hydrophilic and lipophilic drugs [1,2,3]. The stability of the phospholipid bilayer is frequently enhanced by the addition of cholesterol [4]. To increase the circulation time, liposomes are also commonly PEGylated, i.e., polyethene glycol (=PEG) chains are attached to the phospholipid surface thereby inhibiting immune response phagocytosis [5]. Recent studies have also focused on immunoliposomes that bind to antibodies or antibody fragments on their surface, cationic liposomes composed of positively charged phospholipids, and stimuli-responsive vesicles sensitive to local environmental conditions [6,7]

Although more and more liposomal products are entering the phase of clinical trials and registration, the regulation of this area is not complete yet [8]. The liposome-based products belong to the group of non-biologically complex drugs (NBCDs). Due to the complexity and diverse clinical use of NBCDs, it is not possible to establish a general regulatory procedure for these systems; only product-specific guidelines are available [9]. The guideline of the European Medicines Agency [10] provides information on relevant clinical and non-clinical data required for the authorisation of intravenous liposomal products; however, it does not specify concrete analytical and testing strategies or criteria systems, only general principles for the evaluation of the traditional intravenous liposomal products. As even small changes in liposomes significantly affect the result parameters, a well-defined manufacturing process and optimal process control are required to ensure that the quality of the product meets the quality requirements at all times. Creating a surface charge of high absolute value, and thus the production of a long-term stable formulation and recovering the original quality of the freeze-dried samples during reconstitution, are challenging. Although liposome research has a nearly 60-year-old history [11], the proportions of compositions in the literature are still based on traditions, and the liposome recipes have not been optimised in comprehensive studies to date [12,13]. The applied compositions, the chosen production methods, and the opted parameters greatly influence the experimental results. The reason why those formulations were previously studied and how the circumstances were selected is essential for further utilisation of the results. Finding the most appropriate compositions for the purposes and achieving the best results is one of the challenges of this time.

### 1.2. Quality by Design-Based Design and Development

The Quality by Design (QbD) approach [14,15,16,17] and its elements are described in the guidelines of the International Council for Harmonisation of Technical Requirements for Pharmaceuticals for Human Use (ICH) [18,19,20]. Briefly, the steps of a QbD-guided study are to determine the Quality Target Product Profile (QTPP) that describes the essential parameters of the product from the viewpoint of the patient, the clinics and the industry and that in the ideal case should be achieved. The Critical Quality Attributes (CQAs) mean the definitive list of characteristics in the formulation derived from the QTPP and related to the safety and efficacy of the product. The Critical Material Attributes (CMAs) and the Critical Process Parameters (CPPs) are related to the chosen materials and the selected production method. The results of the Risk Assessment (RA) assign the core points of the Design of Experiments (DoE), and the evaluation of the experimental findings leads to the development of the Design Space (DS). The key step of the QbD-driven development process is the RA, which assists in ranking the CQAs and CPPs based on the criticality of their impact on the targeted product quality.

**Figure 1:**
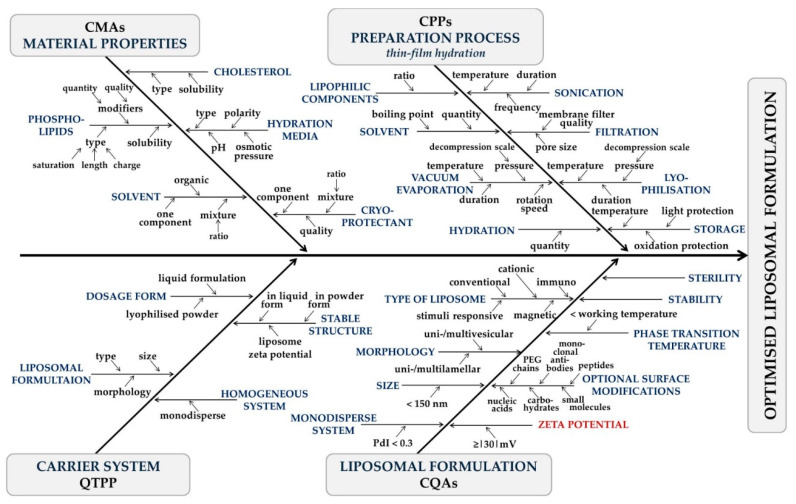
Factors impacting the quality of liposomes made via the thin-film hydration method. Zeta potential plays a crucial role in liposomal formulation through stability criteria. (CMAs = Critical Material Attributes, CPPs = Critical Process Parameters, QTPP = Quality Target Product Profile, CQAs = Critical Quality Attributes).

The effects of factors related to the manufacturing process on product quality are known from a prior study performing an RA. The properties of the liposomes made via thin-film hydration are influenced by the presence and quality of the API, the type and proportion of the wall-forming compounds, the quality of the cryoprotectant and the hydration media, and they are affected by the applied temperature, pressure and settings of the filtration [21]. Based on the results, recommendations are available on the QTPP of an API-free liposomal carrier system [22]; however, the zeta potential needs to be investigated to characterise liposome stability further. The influencing factors on liposome properties as results of the RAs are summarised in Figure 1. The four main sections, i.e., material properties, preparation process-, carrier system-, and liposomal formulation-related factors, correspond to CMAs, CPPs, QTPP, and CQAs, respectively.

### 1.3. Importance of Zeta Potential

Solid surfaces can possess a non-zero surface charge due to dissociative surface groups, specifically adsorbed ions, even without an external potential bias. This so-called surface potential is neutralised by ions of opposite charge attracted to the first layer and in the surroundings of the solid, where the rigid inner Stern layer proceeds in a looser diffuse region. The boundary layer that separates the species attached to the surface and the mobile medium is the slip(ping) plane, generally ~0.2 nm from the surface [23,24,25,26]. The electrokinetic potential, i.e., the zeta potential (ζ) for a colloid system, is the electric potential at the slipping plane relative to a point in the bulk medium away from the surface. It is thus the average electrostatic potential at the hydrodynamic plane of shear [27,28].

Zeta potential characterises the electrical double layer and the nanoparticle, the colloidal formulation itself. It gives information about the stability, circulation time, protein interactions, permeability, and biocompatibility of the nanoparticles [23,25]. Since zeta potential is influenced by temperature, solvent viscosity, pH, ionic strength, and surface characteristics, even minor parameter variations can significantly change its absolute value [23]. The magnitude of the zeta potential can predict the stability of a nanoformulation. High values show highly charged particles that prevent aggregation and ensure redispersion due to repulsive electric forces, while at low zeta potential coagulation may form [29,30,31]. As a general rule, ζ ≥ 30 mV and ≤60 mV in absolute value is considered good and excellent stability, respectively [29,30,32]. Zeta potential ≥ ±30 mV indicates monodisperse formulations without aggregates [26], while ζ ~±20 mV are prone to have only short-term stability, and ζ < 5 mV tends to aggregate rapidly [30]. Nevertheless, the zeta potential value is not the absolute sign of nanoparticle stability. These observations are made for electric stabilisation and low molecular weight surfactants only.

Furthermore, the cellular uptake of nanoparticles is influenced by their shape, size and charge, as their zeta potential affects the cell and tissue binding processes. Higher zeta potentials lead to stronger membrane bindings and a higher level of cellular uptake [28,33]. Moreover, the protein adsorption of the nanoparticles is influenced by electrostatic interactions. Particles with positive zeta potential were found to adsorb well to proteins, while negatively charged ones did not show a significant level of binding. Protein-binding can be influenced by changing the surface charge [30].

The properties of the nano-delivery systems, i.e., circulation, release, and absorption, are also regulated by the characteristics of the nanoparticles [28]. At the liposome–cell interaction, the vesicle wall can adsorb into and fuse with the cell membrane, degrade; and then the released content can diffuse to the cytoplasm. The mechanism of the liposome–cell interaction depends on the features and charge of the liposome surface. From a maximum diameter of 150 nm vesicles, the drug content can be transported into the cell by receptor-mediated endocytosis [7]. Due to the negatively charged endothelial cell surface, tumour cells take up positively charged nanoparticles and retain longer than negative or neutral ones. Other studies showed that particles with a slightly negative zeta potential and a vesicle size of 150 nm are prone to accumulate in tumours. The electrostatic interactions between the nanocarriers and the cell membrane can be utilised for transportation through the blood-brain barrier (BBB). The negatively charged BBB cell membrane attracts particles with positive zeta potential. Reaching a suitable zeta potential is essential for effective nanomedicine, as it affects the targeted therapy, stability and drug release profile [28,30].

### 1.4. Modification of Zeta Potential

Phospholipids are the major components of liposomes and cell membranes. The lipid bilayer is formed due to its amphiphilic property: a hydrophilic ‘head’ (including a phosphate group) and a hydrophobic ‘tail’ (two fatty acid chains) connected via a glycol molecule. Phosphatidylcholine (PC), the most common neutral phospholipid in biological membranes, has a choline molecule in its structure as the ‘head’ group. The stability of the liposomes depends on the lengths and the saturation of the fatty acid chains. The more saturated chains build up the bilayer, the more stable the liposome is. The integrity of the membrane originates from its cholesterol (CH)-content [34]. Zeta potential can be modified by many factors, such as the liposome composition, charged lipids, the pH and the ionic strength of the hydration media, and the production parameters. Charged liposomes can be formed from cationic and anionic phospholipids completing the neutral lipids and causing electrostatic repulsion between the layers [35]. By incorporating various charge-inducing agents into the phospholipid bilayer of the liposome (stearylamine (octadecylamine, SA) or dicetyl phosphate (dihexadecyl phosphate, DCP)), the absolute value of the zeta potential and thus the stability of the vesicles can be increased due to electrostatic interactions [35,36]. SA gives the vesicles a positive/cationic, while DCP a negative/anionic charge, thus preventing aggregation [37]. Experimental results demonstrated the oxidative stability-enhancing effect of these substances as well. Adding cholesterol, SA, and DCP to the composition of the nanocarriers is one of the best practices to improve the stability of the formulations due to the physical stabilisation of the lipid layers [38]. Cationic, synthetic lipids can incorporate positive charges into the liposome membranes and are thus commonly used in nucleic acid delivery [34,39]. SA contains an ionisable nitrogen atom with a positive charge on physiological pH [40]. It distributes asymmetrically in the lipid bilayer, located mainly on the outer surface of the liposomes [41]. Studies on SA-nanoparticles showed increased stability, minimised drug leakage, and a controlled release profile [42]. However, cytotoxicity limits the clinical use of SA as the hydrophilic nitrogen ‘head’ group of the molecule interacts with certain enzymes [36,42]. Other works reported apoptosis induced by SA generating reactive oxygen species, activating protein kinase C, or enhancing the release of apoptosis-dependent proteins, and hemolysis arising from the interaction between the molecule and the negatively charged erythrocyte membrane [43,44]. Human red blood cells are less sensitive to SA; thus, the addition of small amounts can be safe [45]. DCP is a safe cosmetic ingredient, even if it has lower skin permeability than SA due to the negatively charged mammalian skin [36]. Intracerebrally administered SA-liposomes led to respiratory failure and brain damage, while DCP caused epileptic seizures and rapid death in mice [46,47]. A review study made on the immunological and toxicological effects of liposomes concluded that relatively low mol% of cholesterol and PEG is recommended for intravenous application of chemotherapeutic agents. Liposomes with zeta potential less than 30 mV should be considered for gene delivery to minimise toxicities [47]. The toxicity of the formulations can differ based on compositions, delivery routes, and the applied models; thus, they should be evaluated individually in relevant circumstances.

A detailed literature study on previously reported liposomal formulations was performed, and findings on SA and DCP-containing systems were collected in Table 1; Table 2, respectively. The applied ratios and the results varied mainly from study to study, justifying the importance of a time- and material-saving experimental design-based liposome development to optimise the necessary amount of SA and DCP.

In this research, the 3^2^ fractional factorial design was chosen as the material- and time-effective approach to improve vesicle stability through zeta potential optimisation. The goal was to develop formulations with vesicle size under 150 nm, polydispersity index lower than 0.30 and absolute zeta potential higher than 30 mV. For this outcome, parameters determining zeta potentials were identified. Based on the preliminary risk assessment, the ratios between the wall-forming agents (PC, CH) and the charge imparting membrane additives (SA, DCP) affected the liposomal charge and thus were chosen as independent variables.

## 2. Materials and Methods

### 2.1. Materials

Liposomes were made from the following materials: cholesterol (CH) (from Molar Chemicals Kft., Budapest, Hungary); L-α-phosphatidylcholine (1,2-diacyl-sn-glycero-3-phosphocholine) (PC) from egg yolk (60 *w*/*w*% purity); and octadecylamine (=stearylamine, SA), or dihexadecyl phosphate (=dicetylphosphate, DCP) (all purchased from Sigma-Aldrich Chemie GmbH, Munich, Germany). The lipids were dissolved in ethanol 96% (*v/v*) (Molar Chemicals Kft., Budapest, Hungary). Phosphate-buffered saline pH 7.4 (PBS pH 7.4) (ionic strength: 0.16 M), pH 5.6 (PBS pH 5.6) (ionic strength: 0.40 M) and sodium chloride physiological solution (saline solution) (ionic strength: 0.15 M, pH 5.5) were used as hydration media. The composition of these solutions are the followings: *PBS pH 7.4*: 8.0 g/L NaCl, 0.20 g/L KCl, 1.44 g/L Na_2_HPO_4_ × 2 H_2_O, 0.12 g/L KH_2_PO_4_*; PBS pH 5.6*: 0.65 g/L K_2_HPO_4_, 8.57 g/L KH_2_PO_4_; *physiological saline solution*: 9.0 g/L NaCl dissolved in purified water. The undermentioned materials were used to make these hydration media: sodium chloride (NaCl), potassium chloride (KCl), potassium dihydrogen phosphate (KH_2_PO_4_) (Molar Chemicals Kft., Budapest, Hungary), disodium hydrogen phosphate dihydrate (Na_2_HPO_4_ × 2 H_2_O), and dipotassium phosphate (K_2_HPO_4_) (Spektrum-3D Kft., Debrecen, Hungary).

### 2.2. Methods

#### 2.2.1. Factorial Design-Based Experiment Design for Zeta Potential Optimisation

After getting a profound knowledge of the main factors influencing the quality of the liposomal products [24,25], we aimed to prepare stable formulations with zeta potential above 30 mV in absolute value. We have chosen to apply two membrane additives, SA and DCP, and experimentally determine the optimal ratios. The 3^2^ fractional factorial design was used as an experimental design to optimise the zeta potential values. The selected independent variables were the molar quantities of the liposome components: PC, CH, and SA/DCP. As shown in Table 3, these experimental factors were systematically varied at 3 levels and 9 runs in the design. The molar value of PC ranged from 7.5 to 12.5 mmol, of CH from 3.5 to 5.5 mmol, while the amounts of the membrane additives (SA/DCP) were adjusted between 3 and 9 mmol. The optimal component ratios were further investigated by altering the quality of the hydration media. Each composition was prepared in triplicate for parallel measurements. The effects of these independent factors on the vesicle size (Z-average), polydispersity index (PdI) and zeta potential were investigated before lyophilisation. In the case of zeta potential, one-one quadratic response surface was investigated, and the second-order polynomial models were constructed using TIBCO Statistica^®^ 13.4 software (Statsoft Hungary, Budapest, Hungary). The relationship between the variables in the response could be analysed using this second-order Equation:

Y = β_0_ + β_1_x_1_ + β_11_x_1_^2^+ β_2_x_2_ + β_22_x_2_^2^+ β_3_x_3_ + β_33_x_3_^2^, (1)

where Y is the response variable; β_0_ is a constant; β_1_, β_2_, and β_3_ are linear coefficients; and β_11_, β_22_, and β_33_ are quadratic coefficients. The one-way analysis of variance (ANOVA) statistical analysis was carried out to evaluate the significance of the variables. The results were evaluated according to their p-value when variables with p less than 0.05 at a 95% confidence level were considered significant. Response surface plots for zeta potential were plotted according to the regression model for SA/DCP.

#### 2.2.2. Preparation of Liposomes

The liposomes were prepared via the thin-film hydration method [11] with modifications based on our prior findings [21]. The ethanol was evaporated from the alcoholic compositions (Table 3) at 150 mbar and 60 °C in a Rotavapor^®^ R-210/215 (BÜCHI Labortechnik AG, Flawil, Switzerland) rotary evaporator at 25 rpm rotation speed. The lipid film was hydrated, and the formulations were subjected to a 30-min ultrasonication (Elmasonic S 30 H ultrasonic bath (Elma Schmidbauer GmbH, Singen, Germany). The liposomes were vacuum filtered (Rocker 400 oil-free vacuum pump, Rocker Scientific Co., Ltd. New Taipei City, Taiwan) using a 0.45 µm (nylon membrane disk filter 47 mm, Labsystem Kft., Budapest, Hungary), then a 0.22 µm membrane-filter (Ultipor^®^ N66 nylon 6.6 membrane disk filter 47 mm, Pall Corporation, New York, NY, USA). The obtained samples were immediately investigated for vesicle size, polydispersity and zeta potential in a liquid state, then lyophilised for further investigations (SanVac CoolSafe freeze dryer, LaboGene^TM^, Lillerød, Denmark). During lyophilisation, the temperature was gradually decreased from +25 °C to −40 °C at atmospheric pressure, and then the pressure was reduced to 0.01 atm. The samples were dried for 8–10 h before the temperature, and the pressure was increased step by step to +25 °C and 1 atm, respectively. The lyophilised samples were stored at 2–8 °C.

#### 2.2.3. Characterisation of Liposomes

##### Vesicle Size and Zeta Potential Analysis

The vesicle size (expressed in Z-average) and the polydispersity index (PdI) of the liquid liposome formulations were measured using the dynamic light scattering (DLS) technique. The measurements were carried out using a Malvern Zetasizer Nano ZS system (Malvern Panalytical Ltd., Malvern, Worcestershire, UK) equipped with a 633 nm wavelength laser from 1 mL of samples in folded capillary zeta cells (Malvern Panalytical Ltd., Malvern, Worcestershire, UK) at 25 °C. DLS measurements (size, PDI and zeta potential) were performed before lyophilisation and after filtration in all cases.

##### Atomic Force Microscopy (AFM)

Atomic force microscopy (AFM) images of liposomes were obtained under ambient conditions using the tapping mode of an NT-MDT SolverPro Scanning Probe Microscope (NT-MDT, Spectrum Instruments, Moscow, Russia) from one drop of the formulations applied on a freshly cleaved mica surface (Muscovite mica, V-1 quality, Electron Microscopy Sciences, Washington, DC, USA). AFM tips (type PPP-NCHAuD-10, thickness: 4.0 µm, length: 125 µm, width: 30 µm, nominal radius of curvature: 2 nm; NanoWorld AG, Neuchâtel, Switzerland) were applied for the measurements. The non-contact silicon cantilevers had a typical force constant of 42 N/m and a resonance frequency of 330 kHz.

##### Transmission Electron Microscopy (TEM)

The size of the liposomes was determined by transmission electron microscopy (TEM). The TEM images were made with an FEI Tecnai G^2^ X-Twin HRTEM microscope (FEI, Hillsboro, OR, USA) using an accelerating voltage of 200 kV. The TEM measurements were performed after the lyophilisation. Suspensions were prepared from the formulations with ethanol and then dropped onto a carbon film-coated 3 mm diameter copper grid.

##### Thermal Analysis

Differential scanning calorimetry (DSC) measurements (Mettler-Toledo DSC 3^+^ Star^e^ System DSC analyser, Mettler-Toledo International Inc., Columbus, OH, USA) were performed to study the thermodynamic state of the liposomes in the temperature range of 10–65 °C at 2 °C/min heating rate. Phase transition (T_m_) and glass transition (T_g_) temperatures were determined using 6–10 mg of the freeze-dried samples in hermetically sealed aluminium sample pans under a 150 mL/min constant argon flow. Thermogravimetric analysis (TGA) was done in a Mettler-Toledo TGA/DSC 1 thermogravimetric analyser (Mettler-Toledo International Inc., Columbus, OH, USA). In each run, 8–10 mg of the lyophilised samples was heated in aluminium pans at a temperature range of 25–300 °C at a 10 °C/min heating rate, and the mass changes were recorded under dry nitrogen. Empty aluminium pans were used as a blank, and data were normalised to the weight of the sample. The DSC and TGA curves were evaluated using the STAR^e^ 9.30 software (Mettler-Toledo International Inc., Columbus, OH, USA).

##### Fourier Transform Infrared Spectroscopy (FT-IR)

The interactions between the compounds of the liposome were investigated by mid-infrared (MIR) spectroscopy using a Thermo Nicolet Avatar 330 FT-IR spectrometer (Thermo Fisher Scientific, Inc., Waltham, MA, USA). Spectra were recorded on the freeze-dried powder samples in the 4000–400 cm^−1^ wavenumber range with 4 cm^−1^ spectral resolution in absorbance mode. Samples were prepared using a hydraulic tablet press by compressing the lyophilised powders into pellets with potassium bromide (KBr) powder at 10 kN for 2 min (Specac Ltd., Orpington, UK). Pure KBr pellets were used as references.

##### Raman Spectroscopy

Raman spectra were recorded using a Bruker Senterra II Raman microscope (Bruker Scientific Instruments, Billerica, MA, USA) in 180° reflection geometry in the 400–2000 cm^−1^ Raman shift region at 1.5 cm^−1^ resolution. The 785 nm excitation source operated at 50 mW. In each measurement, 5 spectra were averaged with 10 s integration time.

##### Residual Ethanol Measurement via Gas Chromatography-Mass Spectrometry (GC-MS)

The amount of residual ethanol in the samples was determined using a Shimadzu GCMS-QP2010 SE gas chromatograph-mass spectrometer (Shimadzu Corporation, Kyoto, Japan) equipped with a Zebron ZB-5MSi column (Phenomenex, Torrance, CA, USA). The initial oven temperature was 80 °C for 2 min, which was then increased to 180 °C at 20 °C/min and held at 180 °C for 2 more minutes. The mass spectra were recorded in continuous scans from 0.5 to 1.6 min in the 25–46 m/z region. For the measurements, 1 mg/L sample solutions were made in toluene, and 5 μL aliquots were injected in each run. The system was calibrated using a 0.01 mmol/L ethanol solution in toluene.

#### 2.2.4. Physical Stability Studies

The liquid samples were investigated for stability issues via DLS measurements and zeta potential analysis weekly for a month. The physical stability of the freeze-dried nanoparticles was investigated according to the circumstances of the storage conditions described in the ICH Q1A (R2) guideline [73] for accelerated stability tests. The presented results refer to the samples stored at 40 ± 2 °C and 75 ± 5% relative humidity for 3 months. DSC, TGA, FT-IR and Raman studies were done on the stored samples.

#### 2.2.5. Statistical Analysis 

Data analysis and graphs were made in Microsoft^®^ Excel^®^ (Microsoft Office Professional Plus 2013, Microsoft Excel 15.0.5023.100, Microsoft Corporation, Redmond, WA, USA), OriginPro^®^ 8.6 (OriginLab^®^ Corporation, Northampton, MA, USA) and JMP^®^ 13 (SAS Institute, Cary, NC, USA). One-way ANOVA statistical analysis was performed using the TIBCO Statistica^®^ 13.4 software (Statsoft Hungary, Budapest, Hungary). All experiments were performed in triplicates, and the corresponding mean and standard deviations were indicated.

## 3. Results

### 3.1. Factorial Design-Based Experimental Design for Zeta Potential Optimisation

After thoroughly reviewing the available literature on the charge impairing membrane additives (Table 1 and Table 2), the 3^2^ fractional factorial design was chosen for the liposome production optimisation. The molar ratio between the wall-forming lipids (PC, CH) and the special additives (SA/DCP) was examined in the optimisation study. The liposome samples were prepared via the thin-film hydration method with three independent parallels and investigated for the primary outcomes: vesicle size (Z-average), polydispersity index, and zeta potential (collected in Table 4). Only the samples hydrated with PBS pH 5.6 reached the primary set goals; thus, those results are presented in detail (*SA-PBS pH 5.6* and *DCP-PBS pH 5.6* samples).

The results were analysed by the TIBCO Statistica^®^ 13.4 software, and polynomial equations were generated to individually describe the main and the interaction effects of the independent variables on the dependent factor. The relationships between the variables were investigated and described on the zeta potential (Y) according to the ANOVA and regression analysis of the data. As all the size and PdI results of the compositions hydrated with PBS pH 5.6 fulfilled the acceptance criteria of 150 nm and 0.3 PdI, respectively, the impact of the experimental factors was analysed only on the surface charge of the liposomes. Due to the limitations of the factorial plan, the equations provide only approximate results. The material quality limits the nominal maximum point of the response surface curve; thus, the experimental results are expectedly under the predicted values. The importance is in the effect of the coefficients indicating the changes in the responses.

The relationship of the variables on the zeta potential (Y) in the case of the SA-containing formulations could be described with the following Equation:

Y(SA) = 24.622 + 2.633x_1_ + 0.883x_1_^2^ + 0.833x_2_ − 0.967x_2_^2^ − 0.917x_3_ + 0.708x_3_^2^ (2)

The regression coefficient R^2^ = 0.920 showed a good correlation for the surface plot. The molar ratio between PC (x_1_), CH (x_2_) and SA (x_3_) has no significant effect on the surface charge (0.05 < *p*). Zeta potential increases with positive coefficients (x_1_, x_1_^2^, x_2_, x_3_^2^) of the independent variables in Equation (2), while negative coefficients (x_2_^2^, x_3_) have the opposite effect. Liposomes with SA and DCP have positive and negative zeta potential, respectively. The zeta potential (Y) in the DCP-containing formulation is given as follows:

Y(DCP) = −29.833 + 1.250x_1_ − 0.625x_1_^2^ + 0.300x_2_ + 0.650x_2_^2^ − 0.450x_3_ − 0.475x_3_^2^ (3)

A high correlation (R^2^ = 0.984) was found in this case as well. As with the SA formulation, we found no significant effect of the PC (x_1_), CH (x_2_) and DCP (x_3_) molar ratios on zeta potentials (0.05 < *p*). As the DCP liposomes possess a negative charge, the negative coefficients (x_1_^2^, x_3_, x_3_^2^) have favourable effects on the outcome values. Positive coefficients (x_1_, x_2_, x_2_^2^) decrease the absolute zeta potential. 

**Figure 2:**
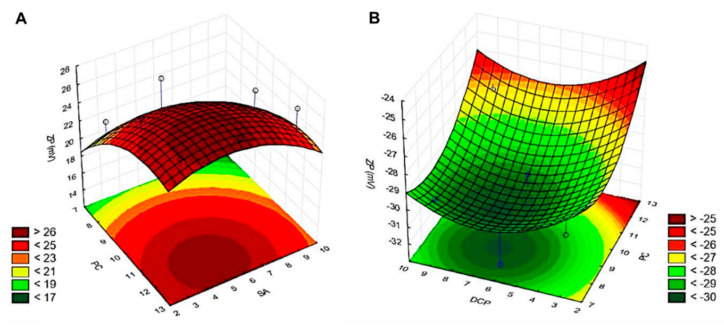
Three-dimensional surface plots of the effect of independent variables on the zeta potential in the 3^2^ fractional factorial design for the compositions made with the membrane additives: stearylamine (**A**) and dicetyl phosphate (**B**) and hydrated with PBS pH 5.6 (PC = L-α-phosphatidylcholine, SA = stearylamine, DCP = dicetyl phosphate).

3D response surface plots visualise the main and interaction effects of two factors at fixed values of the others. The contour plots in Figure 2 show the effect of the PC:SA (A) and PC:DCP (B) molar ratios on the vesicle zeta potential by fixing one variable at a certain level. There is no factor with linearly or quadratically significant effects on zeta potential, and the optimised compositions were deduced from the contour plot of the design space for the SA (dark red) and the DCP (dark green) containing samples: PC:CH:SA = 12.0:5.0:5.0 molar ratio for SA, and PC:CH:DCP = 8.5:4.5:6.5 molar ratio for DCP-liposomes. The resulted liposome forming agent concentrations yielded liposomes where size (<150 nm), polydispersity (PdI < 0.3), and absolute zeta potential (|ζ| > 30 mV) fall in the required parameter regime for the samples hydrated with PBS pH 5.6. The detailed characterisation was done on these optimised formulations.

### 3.2. Characterisation of Liposomes

#### 3.2.1. Vesicle Size and Zeta Potential Analysis

Among the CQAs, the average vesicle size, the polydispersity index and the zeta potential have the highest impacts on the quality of a stable liposome formulation. The optimised ratios between the liposome components were studied by applying PBS pH 5.6, pH 7.4 and saline solution as hydration media (Table 5). The acquired SA-containing samples hydrated with PBS pH 5.6 (*SA-PBS pH 5.6*) and saline solution (*SA-saline sol.*) were significantly smaller (*p* < 0.05 in both cases) than the one made with PBS pH 7.4 (*SA-PBS pH 7.4*) (108 ± 15 nm; 105 ± 18 nm; and 134 ± 24 nm, respectively). Significantly smaller-sized (*p* < 0.05) vesicles were found as well in the case of the DCP-based samples hydrated with PBS pH 5.6 (*DCP-PBS pH 5.6*) (88 ± 14 nm) than with saline solution (*DCP-saline sol.*) (120 ± 10 nm). The uniformity of the vesicles is in the acceptable range for a monodisperse formulation in the case of the lipid-based nanocarrier systems when the PdI value is less or equal to 0.30 [74] and was met in all cases. The zeta potential values were in the acceptance range, higher than 30 mV in absolute value, only in the case when the samples were hydrated with PBS pH 5.6. The measurements indicated significantly more positive (*p* < 0.01 in both cases) zeta potential for the *SA-PBS pH 5.6* sample (+30.1 ± 1.2 mV) than for the *SA-saline sol.* (+13.6 ± 5.3 mV) or the *SA-PBS pH 7.4* (+5.2 ± 2.2) ones. A significant difference (*p* < 0.05) was detected between the zeta potential values presented by the *SA-saline sol.* and the *SA-PBS pH 7.4* samples as well. These values were significantly more negative (*p* < 0.01) in the case of the *DCP-PBS pH 5.6* (−36.7 ± 3.3 mV) sample than in those made with the saline solution (−19.8 ± 2.0 mV) or the PBS pH 7.4 made ones (−19.8 ± 2.0 mV). These results prove the relation between the ionic strength of the hydration media and the zeta potential value of the produced liposomes: the absolute zeta potential value increases with the ionic strength [22,75]. The highest absolute zeta potentials were measured in the samples made with PBS pH 5.6 (ionic strength: 0.40 M).

All of the critical product parameters of the liposomes, such as the vesicle size (108 ± 15 nm; 88 ± 14 nm for the *SA-PBS pH 5.6* and the *DCP-PBS pH 5.6* samples, respectively), the low PdI (0.20 ± 0.04; 0.21 ± 0.02) that indicated monodisperse size distribution and the zeta potential (+30.1 ± 1.2 mV; −36.7 ± 3.3 mV) met the requirements of the nanosized drug delivery systems in the case of the liposomes made with PBS pH 5.6 (Table 5). The positively charged vesicles were larger than the negatively charged counterparts, which can be explained by the spacing difference between the bilayers and the bulkiness of the charge imparting membrane additives [35,48,53]. The *SA-PBS pH 5.6* formulation had significantly larger (*p* < 0.05) vesicles than the *DCP-PBS pH 5.6* ones, while the DCP-containing formulation reached significantly higher (*p* < 0.05) zeta potential in absolute value.

**Figure 3:**
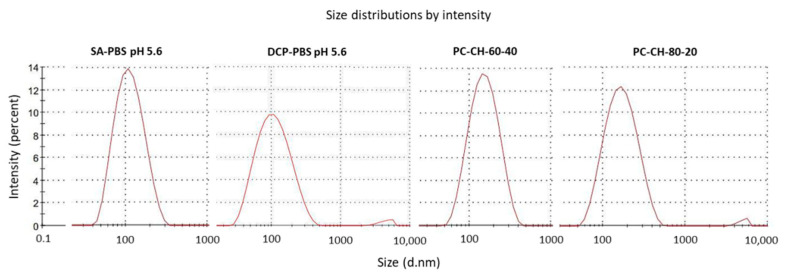
Size distribution results of the SA- and DCP-containing (*SA-PBS pH 5.6*; *DCP-PBS pH 5.6*) and SA- and DCP-free formulations (*PC-CH-60-40*; *PC-CH-80-20*) made with PBS pH 5.6 measured via the dynamic light scattering technique.

Further conclusions could be drawn from examining the formulation compositions. The optimisation was done in molar ratio; using weight ratio allows the direct comparison with previous findings (Table 6). PC:CH = 60:40 and 80:20 weight ratios were found as bests during former research on the topic of optimal phospholipid-cholesterol ratio for liposome formation [21]. The weight ratios of PC in the PC:CH:SA = 12.0:5.0:5.0 and PC:CH:DCP = 8.5:4.5:6.5 molar ratio formulations were essentially the same, i.e., 60 weight units (59.9 and 60.3 in the PC:CH:SA and the PC:CH:DCP compositions, respectively), while 80 weight units were found for the PC:CH ratio alone (82.7 and 78.9 in the PC:CH:SA and the PC:CH:DCP compositions, respectively) in good agreement with the previous results. Investigating the samples made with PBS pH 5.6, the DLS measurements indicated that the addition of SA and DCP to the PC-CH compositions resulted in decreased vesicle size (Figure 3) along with increased zeta potential values: the Tukey inference was *p* < 0.05 for the *SA-PBS pH 5.6* PC-CH-60-40 relationship and *p* < 0.01 for the other relations between the *SA-PBS pH 5.6*, *DCP-PBS pH 5.6*, *PC-CH-60-40* and *PC-CH-80-20* samples. The absolute zeta potential increased significantly with *p* < 0.01 inference for all pairs.

#### 3.2.2. Physical Stability Studies

**Figure 4:**
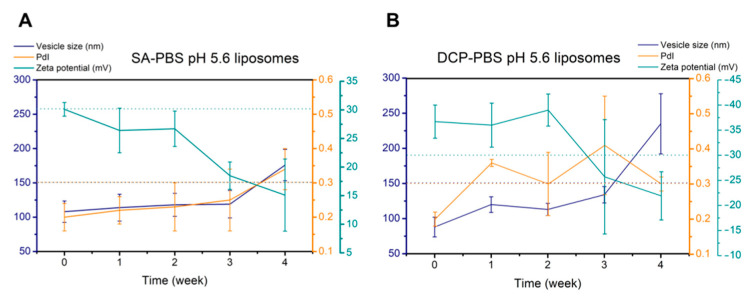
4-week stability tests in the optimised formulations hydrated with PBS pH 5.6 measured by dynamic light scattering: *SA-PBS pH 5.6* (**A**) and *DCP-PBS pH 5.6* (**B**). The changes in vesicle size, polydispersity and zeta potential were followed. Results are from three independent parallels.

Clear formulations were obtained with a blueish opalescence indicating nanoscale colloidal systems. DLS measurements and zeta potential analysis were first done on fresh samples and then repeated weekly for a month. Measurement results are presented in Figure 4. The formulations kept their characteristics in the liquid state within two weeks (results are shown in Table 7); thus, we considered them stable for that period. By the end of the fourth week, the surface charge of the vesicles had significantly decreased (*p* < 0.05 for *SA-PBS pH 5.6* and *p* < 0.01 for *DCP-PBS pH 5.6*). 

Possible changes in the physical stability of the freeze-dried optimised samples made with PBS pH 5.6 were checked after 3 months of storage under the circumstances required for the accelerated stability tests (*SA-PBS pH 5.6-stab.*; *DSC-PBS pH 5.6-stab.*). The performed DSC, TGA, FT-IR and Raman studies indicated stable structures during the investigated period and were presented in detail in the corresponding subsections.

#### 3.2.3. Transmission Electron Microscopy (TEM)

**Figure 5:**
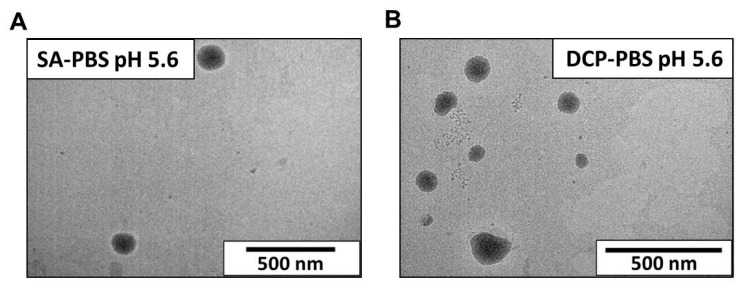
Transmission electron microscopy images of the optimised liposome samples *SA-PBS pH 5.6* (**A**) and *DCP-PBS pH 5.6* (**B**).

Representative TEM images are seen in Figure 5. The average liposome size in the optimised samples hydrated with PBS pH 5.6 is assessable as 100–120 nm.

#### 3.2.4. Atomic Force Microscopy (AFM)

The developed liquid liposomal formulations were converted into a solid phase product via lyophilisation. AFM measurements provided a three-dimensional surface profile of the optimised samples.

**Figure 6:**
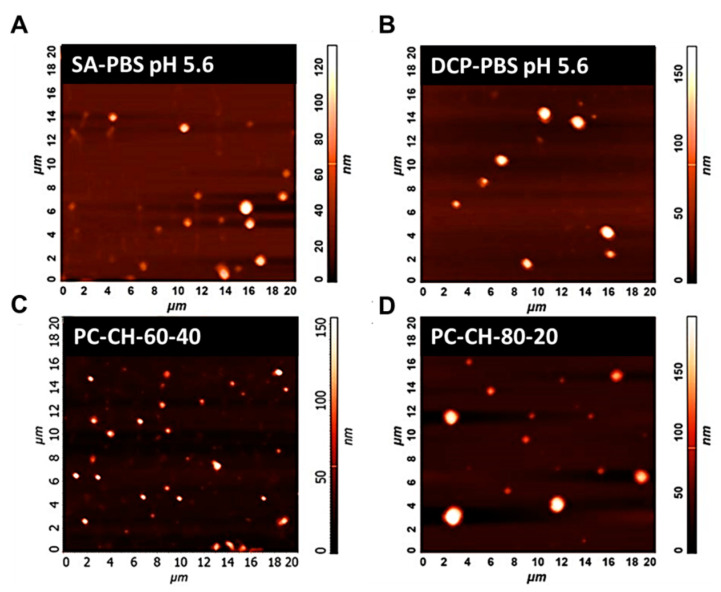
Atomic force microscopy images of the optimised liposome samples *SA-PBS pH 5.6* (**A**) and *DCP-PBS pH 5.6* (**B**) and the membrane additive-free compositions *PC-CH-60-40* (**C**) and *PC-CH-80-20* (**D**).

Figure 6 illustrates the AFM records of the *SA-PBS pH 5.6* (Figure 6A) and the *DCP-PBS pH 5.6* (Figure 6B) samples showing homogeneous size distribution with a mean vesicle size of around 100 nm. The pictures of the membrane additive-free PC- and CH-containing two samples, *PC-CH-60-40* (Figure 6C) and *PC-CH-80-20* (Figure 6D), show larger sizes between 150–180 nm. These images support the information obtained from the DLS measurements.

Based on the AFM measurement results of samples made from the optimal compositions prepared with different hydration media, it can be established that those liposomes are of ~100–110 nm in size with homogeneous size distribution for all samples. These measurement results are in accordance with the results from the DLS measurements.

#### 3.2.5. Thermal Analysis

**Figure 7:**
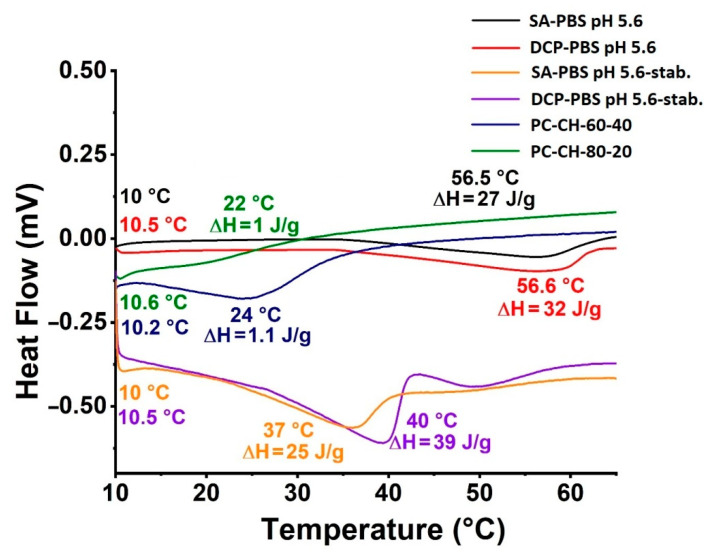
Differential scanning calorimetry results of the optimised liposome samples hydrated with PBS pH 5.6: *SA-PBS pH 5.6* (black) and *DCP-PBS pH 5.6* (red), their results after 3 months in accelerated stability testing circumstances: *SA-PBS pH 5.6-stab.* (yellow) and *DCP-PBS pH 5.6-stab.* (purple), and the membrane additive-free compositions *PC-CH-80-20* (green) and *PC-CH-60-40* (blue).

DSC studies provide information about, among others, the phase transition of the liposomes [76]. The so-called glass transition temperature (T_g_) is an important parameter in characterising the stability of the lyophilised samples [77]. T_m_ and the corresponding enthalpy change (ΔH_m_) influence not only the pharmacokinetics of the pharmaceuticals but the stability of the liposomes as well. High ΔH_m_ implies more rigid phospholipid bilayers [77], while similar transient enthalpies predict a bilayer phase with a similar structure [78]. The calorimetric results (Figure 7) show that the T_g_ for *SA-PBS pH 5.6* (black line) and *DCP-PBS pH 5.6* (red line) was 10 °C and 10.5 °C, respectively. For the membrane additive-free compositions (PC-CH), 10.2 °C (*PC-CH-60-40* (blue line)) and 10.6 °C (*PC-CH-80-20* (green line)) were found. The T_m_ values were 56.5 °C, 56.6 °C, 24.0 °C and 22.0 °C, respectively. The drop in T_m_ on DCP and SA addition is likely caused by the increased distance between the CH-chains due to the intercalation of the compounds, accompanied by the decreasing strength of the van der Waals interactions [79]. Similar ΔH_m_ of 21 J/g and 32 J/g were calculated for the *SA-PBS pH 5.6* and *DCP-PBS pH 5.6* systems, respectively. Compared with the PC-CH formulations (1.1 J/g and 1.0 J/g), these compositions formed more rigid bilayers.

In the case of the stabilised samples, the T_g_ values are the same as those measured in the initial, freshly prepared samples: T_g_ for *SA-PBS pH 5.6-stab.* sample (yellow line) at 10 °C, for the *DCP-PBS pH 5.6* sample (purple line) at 10.5 °C were detected. While the T_m_ values decreased, the ΔH_m_ values remained in the same order of magnitude (25 J/g and 39 J/g), i.e., the stiffness of the double layers lasted for the three months in accelerated stability testing circumstances.

**Figure 8:**
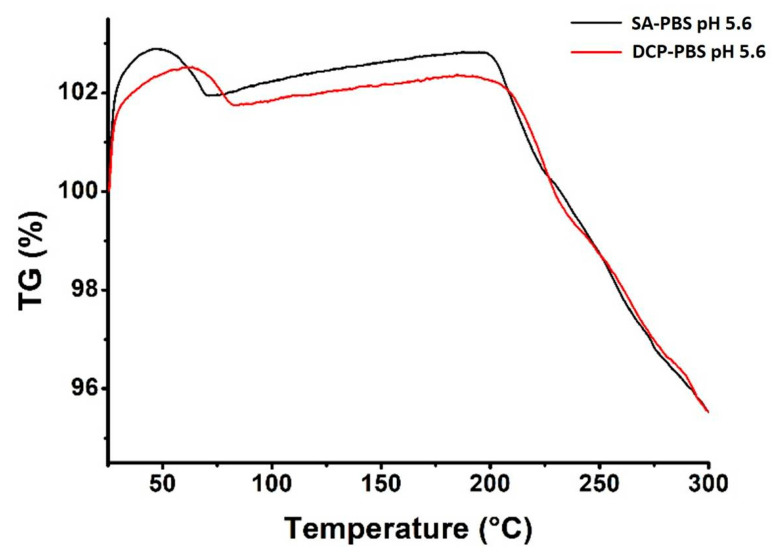
Thermogravimetric analysis of the optimised liposome samples *SA-PBS pH 5.6* (black) and *DCP-PBS pH 5.6* (red).

The thermal stability of the formulations was further investigated via TGA in the 0–300 °C temperature region. Similar gravimetric curves were recorded for both optimised liposomes (Figure 8), and the calculated weight losses are listed in Table 8. The weight loss took place in two steps: The first step at 75–80 °C, indicating the desorption of the physisorbed water content. Due to the minute amount of water in the lyophilised samples, weight losses at around the limit of detection of our system needed to be quantified. Hence the apparent weight gain in the TGA curves. The second step appeared at 200–225 °C, most likely due to molecular changes and chemical degradation in the structures. Both samples suffered ~4% weight loss during annealing. Since degradation occurred at high temperatures only, well above the limit of any practical applications, the optimised formulations are considered stable against temperature during production and storage.

#### 3.2.6. Fourier Transformed Infrared Spectroscopy (FT-IR)

**Figure 9:**
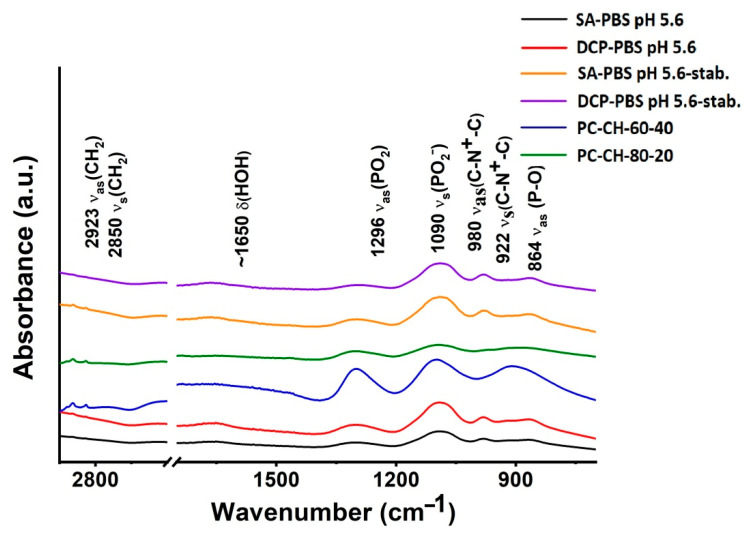
FT-IR spectra of the optimised liposome samples *SA-PBS pH 5.6* (black) and *DCP-PBS pH 5.6* (red), the same samples after 3-month accelerated stability testing *SA-PBS pH 5.6-stab.* (yellow) and *DCP-PBS pH 5.6-stab.* (purple), and the membrane additive-free compositions *PC-CH-60-40* (blue) and *PC-CH-80-20* (green).

Similar FT-IR spectra were recorded for both optimised formulations (Figure 9). Since PC is the wall-forming lipid with the highest concentration in the compositions, its vibrational bands dominate the spectra. Multiple regions can be identified: from 3000 to 2800 cm^−1^ bonds from C-H stretching vibrations can be found, whereas the ~900–600 cm^−1^ regime is the fingerprint region [80]. The former mainly originate from the hydrocarbon chains, while bands corresponding to the vibrations of the polar phospholipid head groups appear at lower wavenumbers (<1800 cm^−1^). At 864 cm^−1,^ the asymmetric ν_as_(P-O), at 922 cm^−1^ the ν_as_^(^N^+^-(CH_3_)_3_), at 980 cm^−1^ the ν_s_(N^+^-(CH_3_)_3_), at 1090 cm^−1^ the symmetric ν_s_((PO)_2_), while at 1296 cm^−1^ the asymmetric ν_as_(PO_2_) stretching can be seen [81]. The symmetric ν_s_(CH_2_) stretchings of the apolar hydrocarbon chains appear at 2850 cm^−1^, while its asymmetric counterpart, ν_as_(CH_2_), is seen at 2923 cm^−1^ [80]. Neither the bands corresponding to SA nor DCP can unambiguously be identified since peaks at 2900–2960, and 2850 cm^−1^ originate from DCP, while vibrations from SA appear at 2920 and 2850 cm^−1^. Thus FT-IR spectra are not conclusive in detecting these charge imparting agents in the formulations.

The FT-IR spectroscopy measurements made on the 3-month accelerated stability test samples (*SA-PBS pH 5.6-stab*. (yellow line); *DCP-PBS pH 5.6-stab.* (purple line)) resulted in the same spectra as the starting samples (*SA-PBS-pH 5.6* (black line); *DCP-PBS pH 5.6* (red line)); no structural change took place.

#### 3.2.7. Raman Spectroscopy

**Figure 10:**
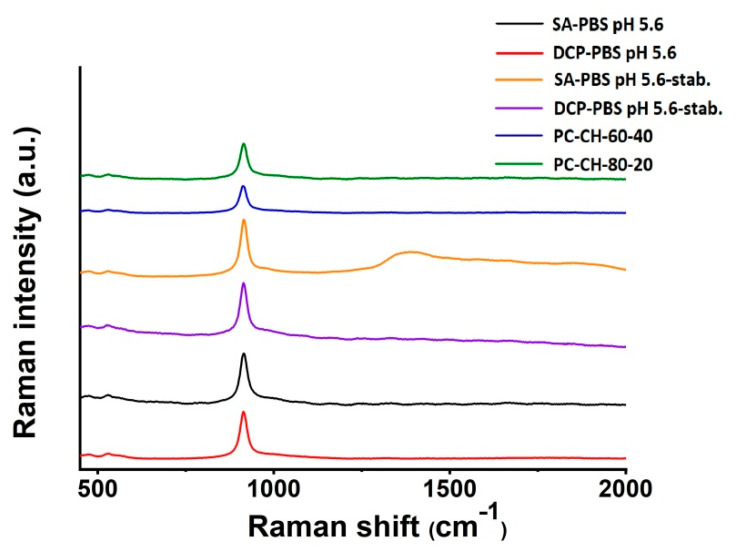
Raman spectra of the optimised liposome samples *SA-PBS pH 5.6* (black) and *DCP-PBS pH 5.6* (red), the same samples after 3-month accelerated stability testing *SA-PBS pH 5.6-stab.* (yellow) and *DCP-PBS pH 5.6-stab.* (purple), and the membrane additive-free compositions *PC-CH-60-40* (blue) and *PC-CH-80-20* (green).

The Raman spectra of the optimised liposomes *SA-PBS pH 5.6* and *DCP-PBS pH 5.6* are compared to those of the membrane additive-free compositions *PC-CH-60-40* and *PC-CH-80-20* in Figure 10. The weak characteristic features of the components match with literature results [82]; however, a new peak at around 914 cm^−1^ Raman shift appears in each spectrum. It might stem from a hitherto unreported interaction between liposome constituents or an unwanted effect of some sample preparation step. To the best of our knowledge, there is no record of such a band in previous studies on PC-CH systems, and hence the investigation of its origin is ongoing.

The Raman spectroscopy measurements made on the 3-month accelerated stability test samples (SA-PBS pH 5.6-stab. (yellow line); DCP-PBS pH 5.6-stab. (purple line)) showed no difference in the spectra compared to the starting samples (SA-PBS-pH 5.6 (black line); DCP-PBS pH 5.6 (red line)), indicating no structural change during the period.

#### 3.2.8. Residual Ethanol Measurement via Gas Chromatography-Mass Spectrometry (GC-MS)

Ethanol is a Class 3 solvent (solvents with low toxic potential) in the ICH guidelines, as it is regarded as less toxic and of lower risk for human health [83]. A daily intake of ≤50 mg, i.e., concentration of 5000 ppm (0.5%), is acceptable in pharmaceuticals. The ethanol residue was quantified via GC-MS, and ethanol concentrations of 11.1 and 23.9 μmol/L (0.51 and 1.10 ppb) were found in the *SA-PBS pH 5.6* and *DCP-PBS pH 5.6* samples, respectively. The amount of remaining ethanol in the samples of *PC-CH-60-40* and *PC-CH-80-20* was below the limit of detection in the current setup.

## 4. Discussion

We would like to point out here that only a few compositions reported in the literature (Table 1 and Table 2) met the expectations of the formulations: vesicle size ≤ 150 nm, PdI ≤ 0.30 and ζ ≥ 30 mV. Mostly one of the three basic characteristic values was not investigated or was out of the acceptance criteria of this study; hence only formulations that lie in the acceptance range are included in this ‘Discussion’ section. Salem et al. and Vhora et al. reached higher zeta potentials than the *SA-PBS pH 5.6* and *DCP-PBS pH 5.6* samples but complemented their formulations with other surfactants and phospholipids. They reached +42.5 ± 2.1 mV and +52.8 ± 3.7 mV zeta potentials with SPC:CH:SA:Span 60 = 1:1:0.15:1 (+flucytosine) and DOPE:SPC:CH:SA = 10:45:29:16 molar ratios, respectively [44,49]. Mishra et al. formulated only SPC- and SA-based (2:0.5 molar ratio) amphotericin B-liposomes without adding cholesterol to the compositions and reached +32.0 ± 0.2 mV with a vesicle size of 140 ± 4 nm [39]. Sharma et al. formulated liposomes with +32.9 ± 2.1 mV of zeta potential from SPC:CH:SA in a 7:3:1.1 molar ratio with a smaller vesicle size (77 ± 2 nm) than *SA-PBS pH 5.6* (108 ± 15 nm) [61]. Inserting monesin as an API into the same liposomes by Rajendran et al. resulted in both higher zeta potential (+43.9 ± 0.9 mV) and vesicle size (121 ± 20 nm) [57]. Salem et al. reached high zeta potential values (−59.1 ± 1.7) by adding Span 60 to the DCP-containing flucytosine liposomes with molar ratios of SPC:CH:DCP:Span 60 = 1:1:0.1:1 [49]. Calvo et al. formulated almost 200 nm large liposomes (195 ± 5 nm) with −47.0 ± 1.0 mV of zeta potential from SPC:CH:DCP = 15:8:1 [70]. Togami et al. reached approximately the same charge, i.e., −49.4 ± 3.5 mV, accompanied by a smaller vesicle size (134 ± 4 nm) in EPC:CH:DCP with a 7:2:1 molar ratio [72]. In evaluation, the modifying effect of the API needs to be considered where it was applied. 

In this study, the effects of these charge imparting membrane additives were investigated in systems without the influence of an API. The necessary PC, CH and SA or DCP molar ratios were determined by applying a 3^2^ fractional factorial design to get liposomal formulations with the predefined CQAs: vesicle size under 150 nm, PdI less than 0.30 and zeta potential higher than |30| mV. The regression models showed no independent variables with a significant effect on the zeta potential; however, the coefficients in the equations describing the relations between the independent variables and the magnitude of the zeta potential predict the changes in its value. The middle points of the design spaces were verified for compliance. The chosen formulations were optimal to meet the requirements of nano-drug delivery systems when the lipid films were hydrated with PBS pH 5.6. The vesicle size was significantly larger (*p* < 0.05) in the case of the *SA-PBS pH 5.6* (108 ± 15 nm) sample than in the *DCP-PBS pH 5.6* (88 ± 14) nm formulation. This phenomenon can be explained by the change in the spacing between bilayers and the bulkiness of the charge imparting membrane additives [35,48,53]. High zeta potentials, typically between 20 and 40 mV, ensure stable systems by decreasing aggregation and increasing polydispersity due to high charge repulsion among liposomes [29]. The absolute zeta potential was significantly higher (*p* < 0.05) in the optimal DCP (−36.7 ± 3.3 mV) than in the SA preparation (+30.1 ± 1.2 mV). The higher repulsive forces between the vesicles resulted in two-week stability in an aqueous medium and up to 3 months in lyophilised form.

The presented results fit into the scientific research area and extend the knowledge on improving liposomal zeta potential. The presented concept helps to establish and perform liposome studies with less effort and more success, and the observations can provide a valuable base for further developments.

## 5. Conclusions

Optimised liposome compositions are vital to achieving highly stable systems for applications in, e.g., targeted drug delivery or diagnostic imaging. The present Quality by Design study is an extension of previous works on the Risk Assessment of liposomes made via the thin-film hydration method. It aims to optimise liposomal formulations through the improvement of the zeta potential of the vesicles, which was modified using stearylamine (SA) or dicetyl phosphate (DCP) charge imparting agents. The Knowledge Space was given about the optimal zeta potentials and membrane additives. A thorough review of the compositions reported in the literature showed that there is no best practice to determine the optimal ratios of the lipid components. Thus, we defined the PC, CH and SA or DCP molar ratios for liposomal formulations with optimised zeta potential and stability via carrying out two 3^2^ fractional factorial designs. The molar ratio of the components was systematically varied 3 levels in 9 runs, and the effect on the vesicle size, PdI, and zeta potential was investigated. Quadratic response surfaces were drawn for the zeta potentials in the case of each charge imparting agent, and the second-order polynomial models describing the effects of the independent variables on the zeta potential were calculated. The optimal molar ratios of the lipids were derived from the contour plots: The optimised compositions for the SA (*SA-PBS pH 5.6*) and DCP (*DCP-PBS pH 5.6*) containing samples turned out to be PC:CH:SA = 12.0:5.0:5.0 and PC:CH:DCP = 8.5:4.5:6.5, respectively. Both formulations met the quality requirements of vesicle size (d(*SA-PBS pH 5.6*) = 108 ± 15 nm and d(*DCP-PBS pH 5.6*) = 88 ± 14 nm), PdI (PdI(*SA-PBS pH 5.6*) = 0.20 ± 0.04 and PdI(*DCP-PBS pH 5.6*) = 0.21 ± 0.02) and zeta potential (ζ(*SA-PBS pH 5.6*) = +30.1 ± 1.2 mV and ζ(*DCP-PBS pH 5.6*) = −36.7 ± 3.3 mV). The high absolute zeta potentials (|ζ| > 30 mV) forecasted long-term stability by reducing vesicle aggregation, and indeed, optimised formulations were stable for up to two weeks in a liquid state. We pointed out that the optimal PC content was ~60 weight% in both *SA-PBS pH 5.6* and *DCP-PBS pH 5.6*, according to prior findings on the charge-inducing agent-free PC:CH system. Since QbD optimisation is an independent method, it supports the latter results on the optimal PC:CH ratio. Moreover, our work provides the parameters to be considered in a QbD-based design for producing liposomes with desired morphology and physical–chemical properties, such as the optimal zeta potential of the vesicles.

## Figures and Tables

**Table 1:** Composition, size, polydispersity index (PdI) and zeta potential of SA-containing formulations (PC = L-α-phosphatidylcholine: EPC = from egg yolk, SPC = from soybean, CH = cholesterol, SA = stearylamine, Span 60 = sorbitan monostearate, Tween 20 = sorbitan monolaurate/Polysorbate 20, DSPC = 1,2-distearoyl-sn-glycero-3-phosphocholine, DOPC = 1,2-dioleoyl-sn-glycero-3-phosphocholine, DOPE = 1,2-dioleoyl-sn-glycero-3-phosphoethanolamine, DSPE-PEG2000 = 1,2-distearoyl-sn-glycero-3-phosphoethanolamine-n-[methoxy(polyethyleneglycol)-2000], GDNF = glial cell-line derived neurotrophic factor).

Composition	Molar Ratio	Drug	Size (nm)	PdI	Zeta Potential (mV)	Source
EPC:CH:SA	1:1:3.85	ketorolac tromethamine	7060 ± -	0.43 ± -	-	Mehanna et al. [48]
SPC:CH:SA:Span 60	1:1:0.15:1	flucytosine	135 ± 12	0.27 ± -	+42.5 ± 2.1	Salem et al. [49]
SPC:SA:Tween 20	20:6.3:2.4	curcumin	252 ± 52	0.17 ± 0.01	+34.0 ± 0.6	Ternullo et al. [50]
SPC:SA:Tween 20	20:6.3:2.4	curcumin	232 ± 68	0.22 ± 0.04	+33.7 ± 1.1	Ternullo et al. [51]
EPC:CH:SA	5.5:1.0: 1.5	butamben	240 ± 65	0.22 ± -	+30.2 ± 3.9	Mura et al. [52]
EPC:CH:SA	6.6:10.3:11.13	sumatriptan	349 ± 100	0.28 ± 0.24	+37.9 ± 3.7	Villasmil-Sánchez et al. [35]
SPC:CH:SA	7:3:1.5	amphotericin B	940 ± 40	-	+28.4 ± 0.3	Soni et al. [53]
SPC:SA	2:0.5	amphotericin B	140 ± 4	0.24 ± 0.04	+32.0 ± 0.2	Mishra et al. [39]
SPC:SA	1:0.5	amphotericin B	202 ± 6	0.39 ± 0.03	+63.0 ± 0.4	Mishra et al. [39]
SPC:CH; SA	9.13:1; 5.18 mg	paclitaxel	193 ± 2	0.17 ± 0.03	+38.2 ± 3.5	Ingle et al. [37]
SPC:CH; SA	7:2; 5.00 mg	resveratrol	146 ± 10	-	+38.0 ±9.1	Jagwani et al. [41]
PC:SA	7:2	doxorubicin	148 ± -	-	+43.1 ± -	De et al. [54]
DSPC:CH:SA	7.5:2.5:0.5	prednisolone, methotrexate	159 ± 2	0.09 ± -	+6.3 ± 0.4	Verma et al. [55]
EPC:CH:SA	7.8:2.6:2.9	pemetrexed disodium	220 ± 5	0.23 ± 0.02	+22.2 ± 0.5	He et al. [45]
SPC:CH:SA	8:1:2	risperidone	209 ± 16	-	+22.4 ± 1.5	Narayan et al. [56]
SPC:CH:SA	8:1:0.25	risperidone	99 ± 7	-	+15.6 ± 1.4	Narayan et al. [56]
SPC:CH:SA	7:3:1.1	monensin	121 ± 20	0.25 ± 0.01	+43.9 ± 0.9	Rajendran et al. [57]
DOPC:CH:SA	10:6:1	GDNF	149 ±11	-	+30.0 ± 3.0	Migliore et al. [58]
DOPC:CH:SA	10:6:1	ovalbumin	299 ± 26	-	+19.0 ± 1.5	Migliore et al. [59]
DOPE:SPC:CH:SA	10:45:29:16	-	95 ± 9	0.24 ± 0.03	+52.8 ± 3.7	Vhora et al. [44]
SPC:CH:SA:DSPE-PEG_2000_	11:7:0.6:1.4	-	209 ± 2	-	+48.7 ± 4.3	Tran et al. [60]
SPC:CH:SA	7:3:1.1	-	77 ± 2	0.21 ± -	+32.9 ± 2.1	Sharma et al. [61]
SPC:SA	7.3:1	-	81 ± 6	0.24 ± 0.02	+17.5 ± 1.8	Caddeo et al. [62]
PC:SA	7:2	-	146 ± -	0.20 ± -	+52.0 ± -	De et al. [63]
SPC:SA	3:1	-	140 ± 49	-	+11.4 ± 0.4	Lotosh et al. [64,65]
EPC:CH:SA	12:5:5	-	108 ± 15	0.20 ± 0.04	+30.1 ± 1.2	***SA-PBS pH 5.6***

**Table 2:** Composition, size, polydispersity index (PdI) and zeta potential of DCP-containing formulations (PC = L-α-phosphatidylcholine: EPC = from egg yolk, SPC = from soybean, CH = cholesterol, DCP = dicetylphosphate, Span 60 = sorbitan monostearate, Tween 80 = sorbitan monooleate/Polysorbate 80, DSPE-PEG2000 = 1,2-distearoyl-sn-glycero-3-phosphoethanolamine-n-[methoxy(polyethyleneglycol)-2000], DPPE = 1,2-dipalmitoyl-sn-glycero-3-phosphoethanolamine, FITC = fluorescein isothiocyanate).

Composition	Molar Ratio	Drug	Size (nm)	PdI	Zeta Potential (mV)	Source
EPC:CH:DCP	1:1:3.85	ketorolac tromethamine	8350 ± -	0.45 ± -	-	Mehanna et al. [48]
PC:CH:DCP	1:1:0.7	tretinoin	318 ± 3	0.43 ± -	−41.2 ± 1.2	Rahman et al. [66]
SPC:CH:DCP	6:1:1.5	silymarin	756 ± -	0.61 ± -	−77.3 ± -	Kumar et al. [67]
EPC:CH:DCP	6.6:10.3:5.49	sumatriptan	549 ± 10	0.37 ± 0.09	−68.1 ± 0.4	Villasmil-Sánchez et al. [35]
SPC:CH:DCP:Span 60	1:1:0.1:1	flucytosine	159 ± 5	0.26 ± -	−59.1 ± 1.7	Salem et al. [49]
SPC:CH:DCP:Tween 80	9:3:1:1	5-fluorouracil	108 ± 11	0.31 ± 0.05	−16.3 ± 1.5	Alomrani et al. [68]
EPC:CH:DCP:DSPE-PEG_2000_:DPPE	7:2:1:1:0.025	FITC-dextran	116 ± -	0.12 ± -	−29.0 ± -	Togami et al. [69]
EPC:CH:DCP:DSPE-PEG_2000_:DPPE	7:2:1:1:0.025	rhodamine B	125 ± -	0.09 ± -	−32.0 ± -	Togami et al. [69]
SPC:CH:DCP: DSPE-PEG_2000_:	11:7:1.4:0.6	-	191 ± 4		−45.1 ± 2.5	Tran et al. [60]
SPC:CH:DCP	15:8:1	-	195 ± 5	0.28 ± -	−47.0 ± 1.0	Calvo et al. [70]
SPC:CH:DCP	10:4:1	-	146 ± 6		−18.6 ± 0.5	Ethemoglu et al. [71]
EPC:CH:DCP	7:2:1	-	134 ± 4	0.12 ± 0.03	−49.4 ± 3.5	Togami et al. [72]
EPC:CH:DCP	8.5:4.5:6.5	-	88 ±1	0.21 ± 0.02	−36.7 ± 3.3	***DCP-PBS pH 5.6***

**Table 3:** Composition of the 3^2^ fractional factorial design with the molar ratio of PC = L-α-phosphatidylcholine, CH = cholesterol, SA = stearylamine or DCP = dicetyl phosphate.

Run	Composition (Molar Ratio)		
	PC	CH	SA/DCP
**1**	7.5	3.5	3.0
**2**	7.5	4.5	9.0
**3**	7.5	5.5	6.0
**4**	10.0	3.5	9.0
**5**	10.0	4.5	6.0
**6**	10.0	5.5	3.0
**7**	12.5	3.5	6.0
**8**	12.5	4.5	3.0
**9**	12.5	5.5	9.0

**Table 4:** Responses of the 3^2^ fractional factorial design studied on liposomes hydrated with PBS pH 5.6 (PC = L-α-phosphatidylcholine, CH = cholesterol, SA = stearylamine, DCP = dicetyl phosphate). Results are expressed in mean ± standard deviation from three independent parallels.

Run	Composition (Molar Ratio)			Responses		
	PC	CH	SA	Vesicle Size (nm)	Polydispersity Index	Zeta Potential (mV)
**1**	7.5	3.5	3.0	121 ± 28	0.22 ± 0.02	+22.0 ± 7.8
**2**	7.5	4.5	9.0	106 ± 21	0.23 ± 0.03	+17.6 ± 3.4
**3**	7.5	5.5	6.0	116 ± 14	0.23 ± 0.02	+24.6 ± 1.4
**4**	10.0	3.5	9.0	93 ± 6	0.22 ± 0.03	+25.0 ± 3.5
**5**	10.0	4.5	6.0	113 ± 16	0.23 ± 0.06	+25.8 ± 3.7
**6**	10.0	5.5	3.0	112 ± 7	0.16 ± 0.01	+26.6. ± 2.7
**7**	12.5	3.5	6.0	111 ± 6	0.19 ± 0.03	+26.3 ± 1.2
**8**	12.5	4.5	3.0	109 ± 7	0.17 ± 0.03	+26.6 ± 0.8
**9**	12.5	5.5	9.0	100 ± 17	0.17 ± 0.01	+27.1 ± 2.8
**Run**	**Composition (molar ratio)**			**Responses**		
	**PC**	**CH**	**DCP**	**Vesicle Size** **(nm)**	**Polydispersity Index**	**Zeta Potential** **(mV)**
**1**	7.5	3.5	3.0	98 ± 11	0.20 ± 0.03	−29.9 ± 1.6
**2**	7.5	4.5	9.0	82 ± 16	0.24 ± 0.02	−29.6 ± 3.4
**3**	7.5	5.5	6.0	108 ± 9	0.21 ± 0.04	−32.5 ± 6,5
**4**	10.0	3.5	9.0	87 ± 16	0.24 ± 0.03	−32.6 ± 2.7
**5**	10.0	4.5	6.0	93 ± 23	0.23 ± 0.03	−29.7 ± 6.2
**6**	10.0	5.5	3.0	119 ± 25	0.21 ± 0.07	−29.7 ± 3.3
**7**	12.5	3.5	6.0	95 ± 8	0.18 ± 0.03	−29.2 ± 3.2
**8**	12.5	4.5	3.0	104 ± 25	0.18 ± 0.02	−27.6 ± 1.3
**9**	12.5	5.5	9.0	105 ± 2	0.18 ± 0.02	−17.7 ± 3.1

**Table 5:** Results of the dynamic light scattering measurements of the optimised formulations (PC = L-α-phosphatidylcholine, CH = cholesterol, SA = stearylamine, DCP = dicetyl phosphate). Results are expressed in mean ± standard deviation from three independent parallels.

Sample	Composition (Molar Ratio)			Responses		
	PC	CH	SA/DCP	Vesicle Size (nm)	Polydispersity Index	Zeta Potential (mV)
SA-PBS pH 5.6	12.0	5.0	5.0	108 ± 15	0.20 ± 0.04	+30.1 ± 1.2
SA-PBS pH 7.4				134 ± 24	0.22 ± 0.07	+5.2 ± 2.2
SA-saline sol.				105 ± 18	0.20 ± 0.10	+13.6 ± 5.3
DCP-PBS pH 5.6	8.5	4.5	6.5	88 ± 14	0.21 ± 0.02	−36.7 ± 3.3
DCP-PBS pH 7.4				112 ± 11	0.22 ± 0.02	−23.5 ± 1.6
DCP-saline sol.				120 ± 10	0.21 ± 0.05	−19.8 ± 2.0

**Table 6:** Results of the dynamic light scattering measurements of the SA-, and DCP-containing and SA-, and DCP-free formulations made with PBS pH 5.6 indicating their compositions (PC = L-α-phosphatidylcholine, CH = cholesterol, SA = stearylamine, DCP = dicetyl phosphate). Results are expressed in mean ± standard deviation from three independent parallels.

	SA-PBS pH 5.6	DCP-PBS pH 5.6	PC-CH-60-40	PC-CH-80-20
	PC:CH:SA	PC:CH:DCP	PC:CH	PC:CH
**Molar ratio**	12.0:5.0:5.0	8.5:4.5:6.5	1:1.32	2.01:1
**Molar % (n/n%)**	54.5:22.75:22.75	43.6:23.1:33.3	43.1:56.9	66.8:33.2
**Weight ratio**	**59.9**:12.5:8.8	**60.3**:16.1:32.8	**60.0**:40.0	80.0:20.0
**Weight % (*w*/*w*%)**	73.8:15.4:10.8	55.2:14.7:30.1	60.0:40.0	80.0:20.0
**PC:CH weight ratio**	**82.7**:17.3	**78.9**:21.1	60.0:40.0	**80.0**:20.0
**Vesicle size (nm)**	108 ± 15	88 ± 14	151 ± 28	172 ± 44
**Polydispersity index**	0.20 ± 0.04	0.21 ± 0.02	0.18 ± 0.07	0.24 ± 0.03
**Zeta potential (mV)**	+30.1 ± 1.2	−36.7 ± 3.3	−9.0 ± 2,4	−8.9 ± 1.3

**Table 7:** 2-week stability tests in the optimised formulations hydrated with PBS pH 5.6 measured by dynamic light scattering. Changes in vesicle size, polydispersity and zeta potential were followed. Results are from three independent parallels.

2-Week Stability	SA-PBS pH 5.6	DCP-PBS pH 5.6
**Vesicle size (nm)**	118 ± 17	113 ± 9
**Polydispersity index**	0.23 ± 0.07	0.30 ± 0.09
**Zeta potential (mV)**	+26.7 ± 3.1	−39.0 ± 3.2

**Table 8:** Optimised formulations (SA = stearylamine, DCP = dicetyl phosphate).

Composition	Starting Point of Weight Loss (°C)	Maximal Weight Loss at 300 °C (%)
**SA-PBS pH 5.6**	75	4
**DCP-PBS pH 5.6**	80	4

## Data Availability

Not applicable.

## Abbreviations

ΔH_m_	enthalpy changes
AFM	atomic force microscopy
ANOVA	analysis of variance
API	active pharmaceutical ingredients
BBB	blood-brain barrier
CH	cholesterol
CMAs	Critical Material Attributes
CPPs	Critical Process Parameters
CQAs	Critical Quality Attributes
DCP	dicetyl phosphate
DLS	dynamic light scattering
DoE	Design of Experiments
DOPC	1,2-dioleoyl-sn-glycero-3-phosphocholine
DOPE	1,2-dioleoyl-sn-glycero-3-phosphoethanolamine
DPPE	1,2-dipalmitoyl-sn-glycero-3-phosphoethanolamine
DS	Design Space
DSC	differential scanning calorimetry
DSPC	1,2-distearoyl-sn-glycero-3-phosphocholine
DSPE-PEG2000	1,2-distearoyl-sn-glycero-3-phosphoethanolamine-n-[methoxy(polyethyleneglycol)-2000]
EMA	European Medicines Agency
EPC	egg phosphatidylcholine
FITC	fluorescein isothiocyanate
FT-IR	Fourier transform infrared spectroscopy
GC-MS	gas chromatography-mass spectrometry
GDNF	glial cell-line derived neurotrophic factor
ICH	International Council for Harmonisation of Technical Requirements for Pharmaceuticals for Human Use
KCl	potassium chloride
K_2_HPO_4_	dipotassium phosphate
KH_2_PO_4_	potassium dihydrogen phosphate
MIR	mid-infrared spectroscopy
Na_2_HPO_4_ × 2 H_2_O	disodium hydrogen phosphate dihydrate
NaCl	sodium chloride
NBCDs	non-biologically complex drugs
PBS pH 5.6	phosphate-buffered saline pH 5.6
PBS pH 7.4	phosphate-buffered saline pH 7.4
PC	L-α-phosphatidylcholine
PdI	polydispersity index
QbD	Quality by Design
QTPP	Quality Target Product Profile
RA	Risk Assessment
RES	reticuloendothelial system
SA	stearylamine
Saline solution	sodium chloride physiological solution
Span 60	sorbitan monostearate
SPC	soy phosphatidylcholine
T_g_	glass transition temperature
TG	thermogravimetric measurements
TGA	thermogravimetric analysis
T_m_	phase transition temperature
Tween 20	sorbitan monolaurate/Polysorbate 20
Tween 80	sorbitan monooleate/Polysorbate 80
